# Enforced BATF expression via clinically approved LNPs enhances adoptive T-cell therapies

**DOI:** 10.1097/BS9.0000000000000291

**Published:** 2026-06-02

**Authors:** Hanchi Ge, Keyu Wang, Lianting Chen, Tong Yin, Fang Zhang, Haoxin Li, Jun Wei, Dan Ding, Wenting Zheng

**Affiliations:** a State Key Laboratory of Experimental Hematology, National Clinical Research Center for Blood Diseases, Institute of Hematology & Blood Diseases Hospital, Chinese Academy of Medical Sciences & Peking Union Medical College, Tianjin 300020, PR China; b Frontiers Science Center for New Organic Matter, State Key Laboratory of Medicinal Chemical Biology, College of Life Sciences and Academy for Advanced Interdisciplinary Studies, Nankai University, Tianjin 300071, PR China

**Keywords:** BATF, CAR-T cell therapy, Lipid nanoparticles

## Abstract

Chimeric antigen receptor (CAR) T-cell therapy is highly effective in hematologic malignancies, yet its durability is limited by insufficient expansion, persistence, and T-cell exhaustion. Basic leucine zipper ATF-like transcription factor (BATF) promotes CD8^+^ T-cell effector differentiation but can drive exhaustion under chronic stimulation. Here, we developed a transient, non-viral strategy to modulate BATF expression in therapeutic T cells using clinically approved lipid nanoparticles (LNPs). Among 3 Food and Drug Administration (FDA)-approved ionizable lipids, SM-102–based LNPs achieved the highest mRNA delivery efficiency in primary T cells. Transient BATF overexpression enhanced T-cell cytotoxicity in vitro without inducing exhaustion. In vivo, BATF mRNA transfection enhanced T-cell expansion, reduced exhaustion, and improved anti-tumor activity for both OT-1 TCR-T cells in melanoma and CD19 CAR-T cells in acute lymphoblastic leukemia. These findings establish a safe and reversible platform for transient transcription factor modulation to optimize T-cell differentiation and function, thereby enhancing the efficacy of adoptive T-cell therapies and supporting clinical translation.

## 1. INTRODUCTION

Adoptive cell therapy (ACT), comprising tumor-infiltrating lymphocytes (TILs), T cell receptor (TCR)–engineered T cells, and chimeric antigen receptor T cells (CAR-T), represents a breakthrough in cancer immunotherapy.^1^ Among these, CAR-T cell therapy has achieved remarkable clinical success, particularly in hematologic malignancies and selected solid tumors.^2^ However, despite high initial response rates, maintaining durable tumor control remains a critical challenge, as more than 50% of patients with hematologic malignancies eventually experience relapse following CAR-T treatment.^3,4^ A primary reason for these failures is the profound dysfunction of transferred T cells within the immunosuppressive tumor microenvironment,^5^ leading to poor tumor infiltration, impaired effector cytokine production, reduced proliferation, limited persistence, and an exhausted phenotype.^6^ Overcoming these barriers is therefore critical to broadening and deepening the efficacy of adoptive T-cell therapy.

T-cell fate, including proliferation, effector differentiation, and exhaustion, is controlled by networks of transcription factors.^7^ Among them, the activator protein 1 (AP-1) family member basic leucine zipper ATF-like transcription factor (BATF) plays a central role in driving CD8^+^ T cells toward an effector state.^8^ Multiple studies demonstrate that BATF is required for proper effector differentiation: BATF cooperates with interferon regulatory factor 4 (IRF4) to drive the transition of precursor exhausted (Tpex) CD8^+^ T cells into effector-like subsets, thereby promoting cytolytic function, cytokine production, tissue infiltration, and proliferative capacity in chronic infection and tumor settings.^9,10^ When BATF is genetically deleted, CD8^+^ T cells fail to form functional effectors, leading to impaired killing and substantially weakened anti-tumor responses.^8,11^ Conversely, controlled BATF overexpression (OE) enhances effector function and anti-tumor activity across multiple tumor models.^10^ However, in tumor microenvironments that drive exhaustion, such as those with persistent antigen exposure or low effector-to-target ratios, prolonged BATF OE can deepen T-cell exhaustion despite promoting an initial effector phenotype.^12^ This dual role highlights that precise, temporal regulation of BATF is critical for optimizing T-cell effector function while avoiding exhaustion.

Achieving such fine-tuned regulation of transcription factors poses significant challenges for current engineering platforms. Strategies based on genomic integration, including viral vector transfection, can work in preclinical settings but carry risks of insertional mutagenesis and uncontrolled long-term expression.^13^ To overcome these limitations, non-viral nanocarriers have been developed to transiently program T cells.^14–16^ Recent studies have utilized polymeric nanoparticles to deliver Foxo1 mRNA for promoting central memory differentiation,^17^ while others employed mPEG-PLGA-PLL polymeric nanoparticles to deliver miR-125a for restoring effector/regulatory T-cell balance in autoimmune disease.^18^ Despite the utility of these materials, lipid nanoparticles (LNPs) distinguish themselves as a superior platform due to their clinically validated safety, high bioavailability, and scalable, simple formulation compared to other nanoparticle classes.^19^ However, to date, the utilization of non-viral nanocarriers for the direct delivery of BATF remains unexplored. Given their favorable biocompatibility and extensive clinical track record, LNPs represent an optimal system to bridge this gap, offering a robust platform for the precise and reversible modulation of BATF in therapeutic T cells.

In this study, we evaluated 3 clinically approved LNPs and identified SM-102–based LNPs (SM-LNPs) as the optimal vehicle for ex vivo mRNA delivery. We demonstrate that transient BATF expression mediated by SM-LNPs reprograms CD8^+^ T-cell differentiation, promoting proliferative and cytotoxic capacities while avoiding exhaustion. Across both mouse OT-1 TCR-T cells in solid tumors and CD19 CAR-T cells in leukemia models, transient BATF induction led to stronger tumor control, improved persistence, and greater polyfunctionality. These findings establish a safe and adjustable strategy using clinically approved LNPs for transient modulation of transcription factors such as BATF, enabling controlled regulation of T-cell differentiation to improve the overall efficacy of ACT products, while offering a rapid path toward clinical application.

## 2. MATERIALS AND METHODS

### 2.1. Mice and cell lines

OT-1 transgenic mice were purchased from The Jackson Laboratory. CAR-T transgenic mice (T cells expressing anti-human CD19 scFv-CD8α-4-1BB-CD3ζ) were generated as previously described.^20^ Sex-matched mice were used at 6 to 10 weeks of age. All animal procedures were conducted in accordance with institutional animal care guidelines and approved by the Institutional Animal Care and Use Committee (IACUC) of the State Key Laboratory of Experimental Hematology (SKLEH), Tianjin, China.

The murine melanoma cell line B16-ovalbumin (OVA) and the human CD19^+^ Arf^−^^/^^−^ BCR-ABL1^+^ progenitor B-ALL cell line (provided by T. Geiger) were cultured in DMEM (Thermo Fisher Scientific, Waltham, Massachusetts) or RPMI-1640 (Thermo Fisher Scientific), respectively, supplemented with 10% fetal bovine serum (FBS; Gibco, Waltham, Massachusetts). Cells were maintained at 37°C in a 5% CO_2_ humidified atmosphere.

### 2.2. LNP synthesis and characterization

LNPs were synthesized using a microfluidic mixing method. The lipid phase was prepared by dissolving the ionizable cationic lipid, DSPC, cholesterol, and DMG-PEG2000 in ethanol at a molar ratio of 50:10:38.5:1.5. For the synthesis of different LNP formulations, the ionizable lipid component was substituted accordingly (DLin-MC3-DMA, SM-102, or ALC-0315) to compare clinically approved formulations.

Messenger RNA (GFP mRNA or BATF mRNA) was dissolved in citrate buffer (pH 4.0, 50 mM) as the aqueous phase. The ethanol and aqueous phases were mixed at a volume ratio of 1:3 (aqueous:ethanol) and a flow rate of 12 mL/min using a microfluidic device, maintaining a nitrogen-to-phosphorus (N/P) ratio of 6:1. Resultant LNPs were centrifuged in ultrafiltration centrifuge tubes (Amicon-Ultra, MWCO 10 KDa), eluted by adding phosphate-buffered saline (PBS) to remove the ethanol, and concentrated to obtain the final LNP.

Particle size, polydispersity index (PDI), and zeta potential were measured using a Zetasizer Nano ZS (Malvern Panalytical, Worcestershire, United Kingdom). Encapsulation efficiency was determined using the RiboGreen RNA assay (Thermo Fisher).

### 2.3. Murine T cell purification and activation

CD8^+^ T cells were isolated from spleens and lymph nodes of C57BL/6, OT-1, or CAR-Tg mice using the MojoSort™ Mouse CD8 Naïve T Cell Isolation Kit (BioLegend, San Diego, California) according to the manufacturer’s instructions. Purified T cells were activated with plate-bound anti-mouse CD3 (5 µg/mL; BioLegend, 100340) and soluble anti-mouse CD28 (5 µg/mL; BioLegend, 102116) in complete RPMI-1640 medium for 20 hours.

### 2.4. T cell transfection with LNPs

Activated T cells were transfected with LNPs in complete RPMI-1640 medium supplemented with recombinant human IL-2 (20 IU/ml; PeproTech, Rocky Hill, New Jersey), human IL-7 (2.5 ng/ml; PeproTech), and IL-15 (25 ng/ml; PeproTech). For transfection optimization, activated murine T cells were treated with LNPs encapsulating GFP mRNA at concentrations of 1, 2, 5, 10, or 20 µg/mL. Transfection efficiency (GFP expression) and cell viability were assessed at 24, 48, and 72 hours post-transfection via flow cytometry. For functional assays, murine T cells (C57BL/6, OT-1, or CAR-T) were activated for 20 hours and then transfected with SM-102 LNPs encapsulating BATF mRNA or empty control LNPs at 10 µg/mL (unless otherwise stated). Cells were maintained in cytokine-supplemented medium and harvested at designated time points for downstream analysis.

### 2.5. In vitro cytotoxicity assay

Target cells (B16-OVA for OT-1 T cells; hCD19^+^ B-ALL for CAR-T cells) were co-cultured with LNP-transfected effector T cells (BATF-OE or Control) at effector-to-target (*E*:*T*) ratios of 1:1, 5:1, and 10:1 in 48-well plates. After 24 or 48 hours of co-culture, the absolute number of viable target cells was quantified using flow cytometry with 7-aminoactinomycin D (7-AAD; BioLegend, A9400) exclusion and Precision Count Beads (BioLegend, 424902). T cell expansion during co-culture was simultaneously assessed by counting CD8^+^ T cells. Effector cell activation status was determined through surface marker profiling.

### 2.6. In vivo B16-OVA solid tumor model

For survival studies, C57BL/6 mice were subcutaneously inoculated in the flank with 0.5 × 10^6^ B16-OVA melanoma cells. When tumors reached approximately 2 × 3 mm, mice received an adoptive transfer of 2 × 10^6^ OT-1 CD8^+^ T cells that had been transfected ex vivo with BATF-LNP or control-LNP. Tumor volumes were calculated by the formula: length × width × ([length × width]^0.5^) × π/6. Survival was monitored every 2 days, with endpoints defined as tumor volume >1500 mm^3^ or ulceration.

For phenotypic analyses, mice were implanted with 1 × 10^6^ B16-OVA cells and monitored until tumors reached approximately 7 × 7 mm, at which point they received 2 × 10^6^ LNP-transfected OT-1 T cells intravenously. On day 5 post-transfer, tumors and tumor-dLNs were harvested. Tumors were digested with 0.5 mg/mL collagenase IV (Worthington, LS004188, Lakewood, New Jersey) and 50 IU/mL DNase I (Roche, 10104159001, Basel, Switzerland) for 1 hour at 37°C, followed by filtration through 70-μm strainers. TILs were isolated by density-gradient centrifugation over Percoll (GE, 17-0891-01, Chicago, Illinois). Single-cell suspensions were used directly for staining.

### 2.7. In vivo B-ALL leukemia model

For survival studies, C57BL/6 mice were intravenously injected with 0.5 × 10^6^ hCD19^+^ Arf^−^^/^^−^ BCR-ABL1^+^ pre–B ALL cells. On day 5 after leukemia inoculation, mice received an adoptive transfer of 2 × 10^6^ CAR-T cells transfected ex vivo with BATF-LNP or control-LNP. Natural mortality of the animals was used as the endpoint for Kaplan–Meier survival analysis.

For mechanistic studies, mice were injected intravenously with 1 × 10^6^ leukemic cells and treated on day 6 post-inoculation with 2 × 10^6^ CAR-T cells that had been transfected with either BATF-LNP or Control-LNP. On day 5 post–T-cell infusion, bone marrow and spleens were collected. Bone marrow cells were filtered through 70-µm strainers. Spleens were mechanically disrupted, and the resulting cell suspensions were treated with ACK lysis buffer to remove red blood cells (RBCs) and then filtered through a cell strainer. All cell samples were resuspended in PBS supplemented with 2% FBS for immediate flow cytometry analysis.

### 2.8. Flow cytometry and antibodies

Samples were acquired on an Agilent NovoCyte Quanteon and analyzed with FlowJo v10.8+. Surface staining was performed for 25 minutes at 4°C in PBS containing 2% FBS. For intracellular transcription factors (BATF, Ki67, TOX, TCF1), cells were fixed with the Foxp3/Transcription Factor Staining Buffer Set (eBioscience, 00-5523-00, San Diego, California). Cytokine and granzyme B staining was performed after 4 hours ex vivo restimulation with Cell Activation Cocktail (containing PMA/ionomycin) plus Brefeldin A (BioLegend, 423304) using IC Fixation Buffer (eBioscience, 00-8222-49) and permeabilized with Permeabilization Buffer (Invitrogen, 00-8333-56, Waltham, Massachusetts) following the manufacturer’s protocol. The following antibodies were used: Ki67 (eBioscience, 404-5698-82), CD25 (BioLegend, 102026), CD44 (BioLegend, 103006), CD62L (eBioscience, 12-0621-83), PD-1 (BioLegend, 135224), TIM-3 (eBioscience, 25-5870-82), BATF (CST, 27120S, Danvers, Massachusetts), CD45.1 (BioLegend, 110743), TCF1 (CST, 14456S), TOX (eBioscience, 12-6502-82), IL-2 (eBioscience, 12-7021-82), TNF-α (eBioscience, 506344), IFN-γ (eBioscience, 45-7311-82), GZMB (BioLegend, 372206), CD90.1 (eBioscience, 64-0900-82).

### 2.9. Statistical analysis

Statistical analyses were performed using GraphPad Prism software. Comparisons between 2 groups were analyzed using 2-tailed unpaired Student *t* tests. For the in vitro killing assay, the data were analyzed using 2-way analysis of variance (ANOVA), followed by appropriate multiple comparison tests. Tumor growth curves were analyzed using 2-way ANOVA, and survival curves were compared using the log-rank (Mantel–Cox) test. Data are presented as mean ± standard error of the mean (SEM). *p* < 0.050 was considered statistically significant.

## 3. RESULTS

### 3.1. Comparison of mRNA transfection efficiency in T cells using approved LNPs

**Figure 1.**
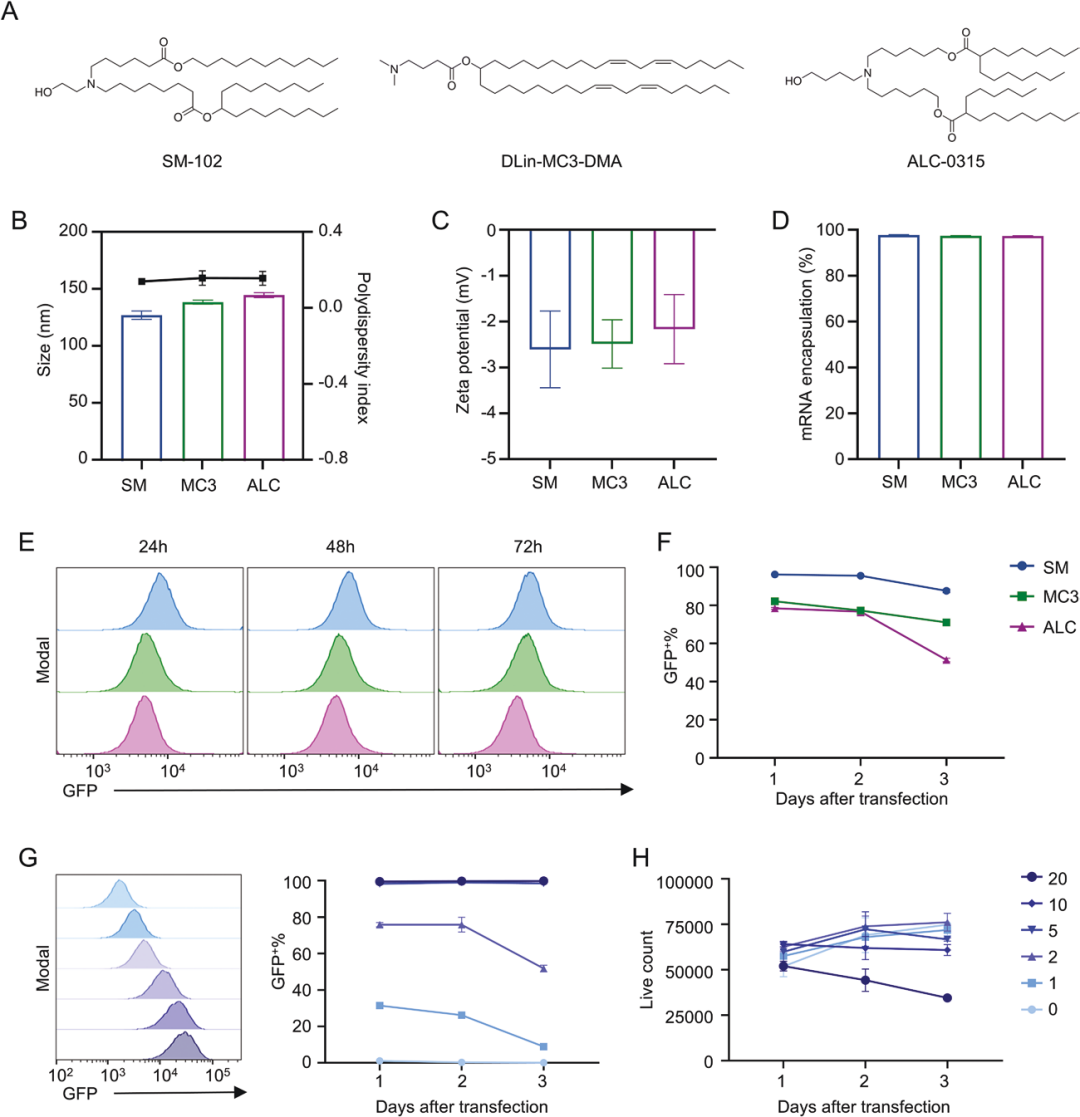
Comparison of mRNA transfection efficiency in T cells using clinically approved commercial LNPs. (A) Chemical structures of DLin-MC3-DMA, SM-102, and ALC-0315. Structures were drawn using ChemDraw software. (B–D) Physicochemical characterization of the indicated LNPs formulations. Quantification of particle size (mean diameter), PDI (B), zeta potential (mV) (C), and mRNA encapsulation efficiency (D). (E) Representative flow cytometry histograms showing GFP expression in primary mouse T cells at 24, 48, and 72 h after transfection with SM-LNPs, MC3-LNPs, or ALC-LNPs encapsulating GFP mRNA. (F) Kinetic analysis of GFP expression in primary mouse T cells corresponding to the histograms shown in (E). Data are presented as the percentage of GFP^+^ cells. (G) Optimization of LNP dosage in primary T cells. Dose-dependent GFP expression in mouse CD8 T cells at days 1, 2, and 3 post-transfection with SM-LNPs at the indicated mRNA concentrations (0–20 µg/mL). (H) Analysis of cytotoxicity following LNP transfection. Cell count of mouse primary T cells post-transfection with SM-LNPs at the indicated concentrations. Data in (B, C, D, F, G, and H) are presented as the mean ± SEM (n = 3). ALC-LNPs = ALC-0315–based lipid nanoparticles, GFP = green fluorescent protein, LNPs = lipid nanoparticles, MC3-LNPs = DLin-MC3-DMA–based lipid nanoparticles, PDI = polydispersity index, SM-LNPs = SM-102–based LNP.

Given the wide range of nanoparticle platforms available for nucleic acid delivery, we chose LNPs for T-cell transfection because of their clinically validated safety, favorable biocompatibility, and scalable manufacturing.^19^ To enable rapid clinical application, we focused on 3 ionizable lipids already used in approved LNP-based products: DLin-MC3-DMA (used in Onpattro), SM-102 (used in Moderna’s mRNA-1273 vaccine), and ALC-0315 (used in Pfizer/BioNTech’s BNT162b2 vaccine) (**Fig. 1A**).^21^ The LNPs exhibited an average particle size of approximately 100 nm with a low PDI (<0.2), indicating uniform distribution (**Fig. 1B**). The zeta potentials were near neutral, varying between −1.3 and −3.4 mV, consistent with the expected surface charge range for ionizable lipid-based formulations (**Fig. 1C**). Moreover, all formulations exhibited high mRNA encapsulation efficiencies exceeding 97% (**Fig. 1D**). These data demonstrate that all LNP formulations were well-formed, stable, and suitable for biological transfection applications.

We next compared the ability of these LNPs to deliver mRNA to primary T cells. mRNA encoding green fluorescent protein (GFP) was encapsulated as a reporter and used to transfect CD8^+^ T cells isolated from C57BL/6 mouse spleen and lymph nodes. Flow cytometric analysis at 24, 48, and 72 hours revealed that SM-LNPs consistently achieved the highest transfection efficiency across all time points. At the 24-hour peak, SM-LNPs achieved almost complete transfection, with 96.17 ± 0.38% of CD8^+^ T cells expressing GFP. In comparison, DLin-MC3-DMA–based LNPs (MC3-LNPs) and ALC-0315–based LNPs (ALC-LNPs) reached 82.13 ± 2.06% and 78.47 ± 0.84% GFP^+^ cells, respectively (**Fig. 1E** and F). These results demonstrate that SM-LNPs provide stronger reporter protein expression in primary T cells compared to the other clinically approved formulations.

Based on the superior functional performance of SM-LNPs, we next optimized their ex vivo dose to identify conditions suitable for T-cell manufacturing. Primary T cells were treated with SM-LNPs co-formulated with GFP mRNA and the fluorescent tracer DiI over a range of concentrations and analyzed at 24, 48, and 72 hours post-transfection. GFP mean fluorescence intensity (MFI) increased in a dose-dependent manner, and the percentage of GFP^+^ cells reached a stable level at concentrations ≥5 µg/mL, with >99% of cells expressing GFP (**Fig. 1G** and Figure S1A, https://links.lww.com/BS/A153). DiI fluorescence correlated with GFP expression, confirming efficient LNP uptake across these doses (Figure S1B, https://links.lww.com/BS/A153). All concentrations up to 10 µg/mL maintained high live cell counts, comparable to untreated controls (**Fig. 1H**), whereas 20 µg/mL produced a clear loss of viability, indicating high-dose–associated cytotoxicity (**Fig. 1H**).

These results demonstrate that SM-LNPs show higher transfection efficiency in primary T cells than other clinically approved ionizable lipid formulations, and efficiently transfect primary T cells across a broad dose range, with 5 to 10 µg/mL achieving robust expression while maintaining viability. Therefore, SM-LNPs were used at 5 and 10 µg/mL for the initial functional mRNA delivery experiment.

### 3.2. BATF OE reprograms T cell differentiation and proliferation

**Figure 2.**
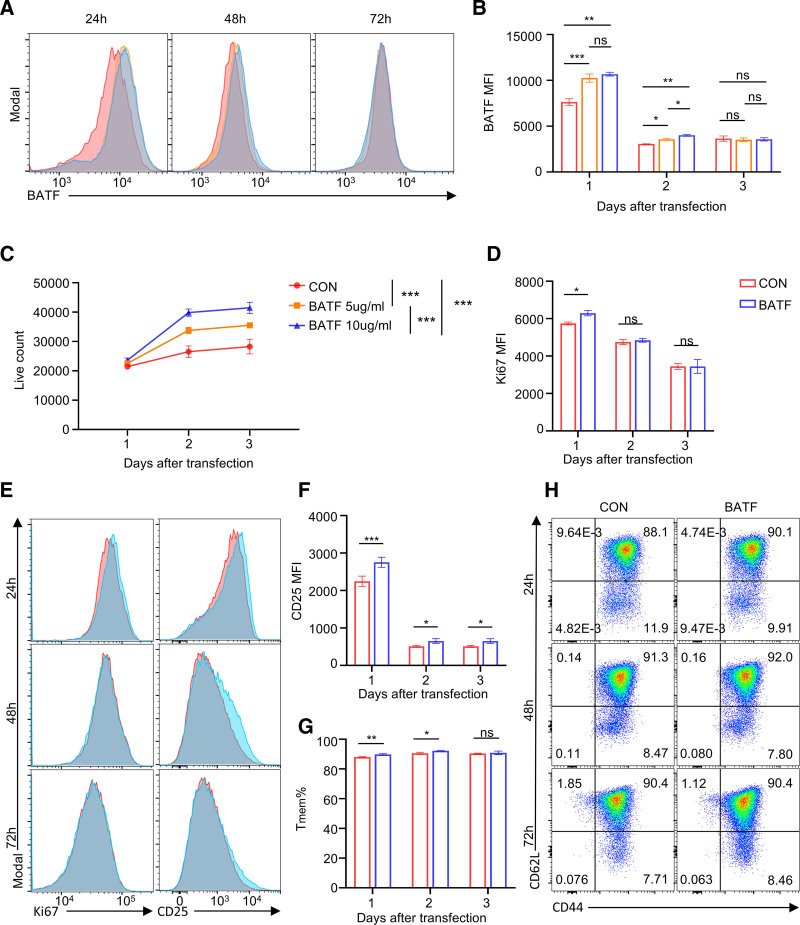
BATF overexpression reprograms T cell differentiation and proliferation. (A) Representative flow cytometry histograms showing intracellular BATF expression in primary mouse T cells 24, 48, and 72 h after transfection with control-PBS or BATF-LNP (SM-102, 5 or 10 µg/mL). (B) Kinetic analysis of BATF protein expression (MFI) in T cells at 24, 48, and 72 h post-transfection. (C) Quantification of viable T cell numbers at indicated time points following transfection with 5 or 10 µg/mL BATF mRNA, showing dose-dependent proliferation. (D) Quantification of Ki67 MFI corresponding to the histograms shown in (E). (E) Flow cytometry histograms of intracellular Ki67 (left) and CD25 (right) staining in T cells at 24, 48, and 72 h post-transfection with control-PBS or BATF-LNP (SM-102, 10 µg/mL). (F) Quantification of CD25 MFI corresponding to the histograms shown in the panel. (G) Quantification of the percentage of memory T cells (Tmem; CD62L^+^CD44^+^) among CD8^+^ T cells. (H) Representative flow cytometry plots showing the expression of memory markers CD44/CD62L on T cells following BATF overexpression. Data in (B, C, D, F, and G) are presented as the mean ± SEM. *p* values were determined by an unpaired 2-tailed Student *t* test.*p < 0.050, **p < 0.010, ***p < .001. BATF = basic leucine zipper ATF-like transcription factor, LNPs = lipid nanoparticles, MFI = mean fluorescence intensity, ns = not significant, PBS = phosphate-buffered saline, SEM = standard error of the mean.

Given prior work showing that BATF acts as a pioneer transcription factor controlling early effector and memory CD8^+^ T cell programs in cooperation with IRF4,^8,10^ we wondered whether transient OE of BATF could regulate the fate of T cells in vitro. Primary mouse T cells were activated with anti-CD3/CD28 for 24 hours and transfected with BATF mRNA-loaded SM-102-LNPs at 5 or 10 µg/mL. Transfection efficiency was confirmed by flow cytometry via intracellular BATF staining, revealing approximately a 1.34-fold increase of BATF protein compared to PBS-treated controls on day 1 post-transfection (**Fig. 2A and B**). BATF expression peaked at 24 hours and gradually returned to baseline by 48 to 72 hours (**Fig. 2B**), consistent with the transient kinetics of mRNA–LNP–mediated expression. These observations confirm efficient and transient BATF upregulation via mRNA-LNPs.

To assess the impact of BATF OE on T cell expansion, we monitored viable cell counts and cell cycle marker Ki67 expression following LNP-BATF delivery. Viable cell counts showed a clear dose-dependent expansion, with 5 µg/mL BATF mRNA resulting in a 25.65% increase in total cell numbers on day 3 post-transfection, whereas 10 µg/mL led to a greater 46.66% increase on day 3 (**Fig. 2C**) upon TCR stimulation. Accordingly, we used SM-LNPs at 10 µg/mL to deliver functional mRNA into primary T cells in subsequent experiments. Consistently, intracellular Ki67 staining revealed a significant increase in Ki67 MFI relative to PBS-treated controls on day 1 post-transfection, indicating accelerated cell cycling (**Fig. 2D and E**). Together, these results demonstrate that transient BATF OE robustly promotes T cell expansion without compromising viability.

We next examined BATF OE T cells for effects on T cell activation and exhaustion. BATF OE led to a notable increase in CD25 expression, with CD25 MFI elevated by 25.54% relative to controls, reflecting enhanced T cell activation (**Fig. 2E and F**). Notably, transient BATF OE did not alter the exhaustion status of T cells as the exhaustion-associated markers PD-1 or TIM-3 levels remained unchanged (Figure S2A–C, https://links.lww.com/BS/A153). Interestingly, BATF OE was associated with a modest but statistically significant increase in the proportion of CD44^+^CD62L^+^ T cells, suggesting a shift toward a memory-like phenotype (**Fig. 2G and H**; Figure S2D and E, https://links.lww.com/BS/A153). This observation is in line with previous studies reporting a role for BATF–IRF4–dependent transcriptional programs in supporting clonal expansion and memory CD8^+^ T cell responses under certain contexts.^8,22^

Collectively, these findings show that during T-cell culture, transient BATF OE contributes to activation and proliferation of T cells, and slightly enriches a memory-like surface phenotype, without induction of exhaustion. These data support the use of 10 µg/mL BATF-LNPs as a working dose for downstream assays examining BATF-mediated enhancement of T cell function.

### 3.3. BATF OE enhances T cell cytotoxicity in vitro

**Figure 3.**
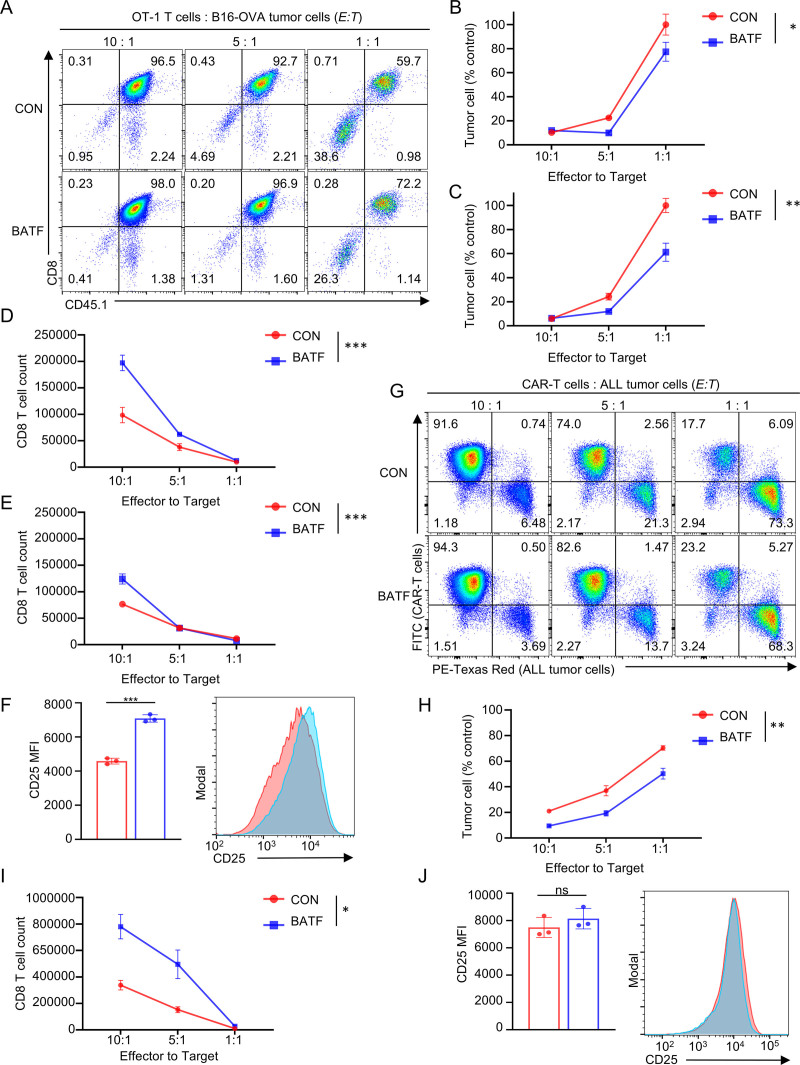
BATF overexpression enhances T cell cytotoxicity in vitro. (A) Representative flow cytometry plots showing B16-OVA tumor cell killing by OT-1 T cells at different effector-to-target (*E*:*T*) ratios after 24 h of co-culture. (B and C) Quantification of viable B16-OVA tumor cells after 24 h (B) and 48 h (C) of co-culture with OT-1 T cells (transfected with control-LNP or BATF-LNP) at the indicated effector-to-target (*E*:*T*) ratios. Data are presented as percentage relative to controls without OT-1 T cells. (D and E) Quantification of absolute OT-1 T cell numbers during 24 h (D) and 48 h (E) co-culture at the indicated *E*:*T* ratios. (F) Analysis of CD25 expression on OT-1 T cells after 24 h co-culture with B16-OVA cells (*E*:*T* = 10:1). (G) Representative flow cytometry plots showing ALL tumor cell killing. RFP-expressing hCD19^+^ ALL cells (detected in the PE-Texas Red channel) were co-cultured with FITC-labeled CAR-T cells at indicated *E*:*T* ratios (10:1, 5:1, 1:1) for 24 h. (H) Quantification of viable ALL tumor cells after 24 h co-culture with CAR-T cells at the indicated *E*:*T* ratios. Data presented as percentage relative to controls without CAR-T cells. (I) Quantification of absolute CAR-T cell numbers during 48 h co-culture at the indicated *E*:*T* ratios. (J) Quantification of CD25 MFI on CAR-T cells after 48 h of co-culture (*E*:*T* = 5:1). Data are presented as the mean ± SEM (n = 3). *p* values were determined by 2-way ANOVA test. *p < 0.050, **p < 0.010, ***p < .001. ALL = acute lymphoblastic leukemia, ANOVA = analysis of variance, BATF = basic leucine zipper ATF-like transcription factor, CAR-T = chimeric antigen receptor T cells, FITC = fluorescein isothiocyanate, LNPs = lipid nanoparticles, MFI = mean fluorescence intensity, OVA = ovalbumin, RFP = red fluorescent protein, SEM = standard error of the mean.

We next evaluated how transient BATF OE influences T cell cytotoxicity in vitro with different tumor immunotherapy models. In the OT-1 system, mouse OVA-specific CD8^+^ T cells were activated for 24 hours, transfected with BATF mRNA–loaded SM-LNPs (10 µg/mL), and co-cultured with OVA-expressing B16 melanoma cells (B16-OVA). BATF-overexpressing OT-1 cells mediated stronger tumor cell killing than empty LNP–treated controls at both 24 and 48 hours and across multiple *E*:*T* ratios (**Fig. 3A–C**). At a 1:1 *E*:*T* ratio, BATF OE increased killing efficacy to 22.6% at 24 hours and 38.9% at 48 hours, indicating enhanced cytotoxic activity relative to control OT-1 T cells.

BATF-transfected T cells also exhibited greater expansion upon tumor-specific antigen stimulation, reaching up to 2-fold higher cell counts at the 10:1 *E*:*T* ratio at 24 hours (**Fig. 3D and E**). Flow cytometric analysis of live CD8^+^ T cells revealed a 1.55-fold increase in CD25 MFI (control: 4584 vs. BATF: 7084; **Fig. 3F**), indicating better activation.

A parallel enhancement in anti-tumor activity was observed in the mouse CAR-T model engineered with the FMC63-derived anti-human CD19 CAR. When co-cultured with hCD19^+^ Arf^−^^/^^−^ BCR-ABL pre-B acute lymphoblastic leukemia (pre-B ALL) cells,^20^ BATF OE CAR-T cells achieved better tumor control at 24 hours than control CAR-T cells over all *E*:*T* ratios examined (**Fig. 3G and H**). Consistent with the OT-1 system, BATF-transfected CAR-T cells also demonstrated significantly greater numerical expansion during the 48-hour co-culture, with 2.30-fold at 10:1 and 3.24-fold at 5:1 *E*:*T* (**Fig. 3I**). CD25 expression on CAR-T cells was similar between BATF OE and control groups under these conditions (**Fig. 3J**).

Together, these data show that SM-LNP–mediated transient BATF OE enhances CD8^+^ T cell cytotoxicity and expansion in vitro in both TCR- and CAR-based models. More importantly, BATF-LNP–treated T cells exhibited more efficient anti-tumor activity in vitro without requiring permanent genetic modification.

### 3.4. Transient BATF mRNA OE enhances the anti-tumor efficacy of TCR-T cells in solid tumors

**Figure 4.**
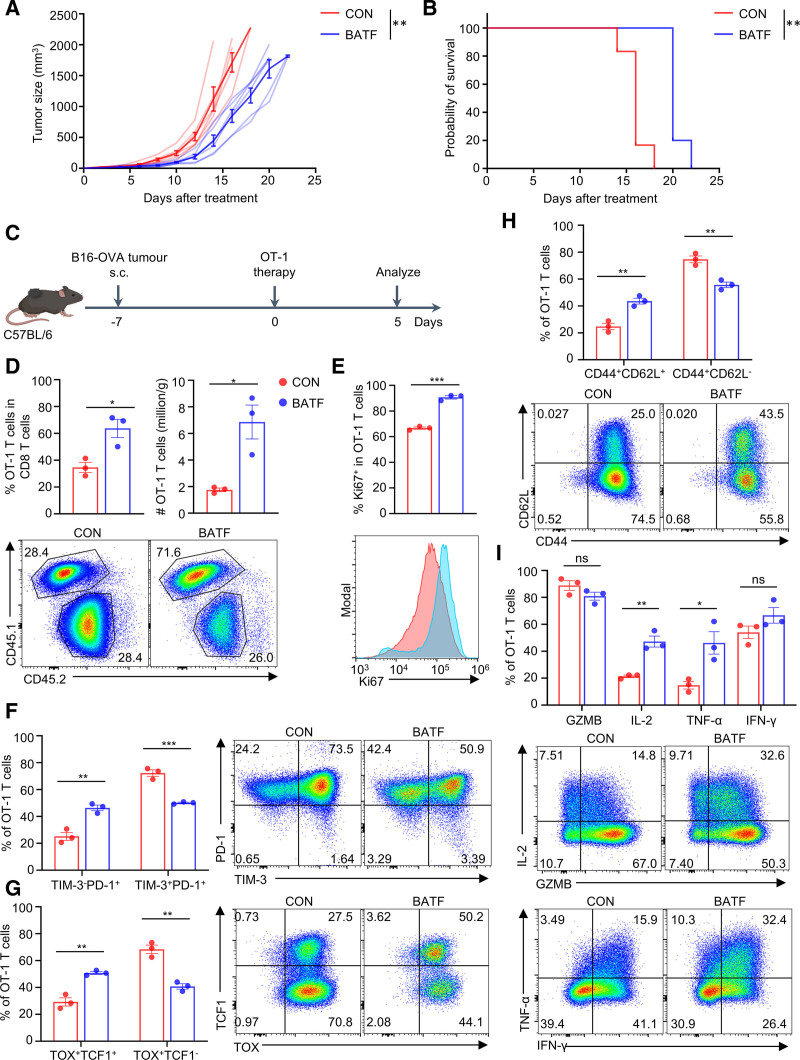
Transient BATF mRNA overexpression enhances the anti-tumor efficacy of TCR-T cells in solid tumors. (A) Tumor growth curves of C57BL/6 mice bearing B16-OVA tumors treated with adoptive transfer of OT-1 T cells transfected with control-LNP or BATF-LNP. (B) Kaplan-Meier survival curves of tumor-bearing mice from (A). (C) Schematic of the in vivo mechanistic study analyzing TILs and dLNs at day 5 post-adoptive transfer. (D) Quantification of donor OT-1 T cell infiltration in tumors. Bar graphs show the percentage of donor cells among total CD8^+^ TILs (left) and the absolute number of donor OT-1 cells per gram of tumor (right). (E) Quantification of the percentage of Ki67^+^ cells among donor OT-1 TILs. (F) Quantification and representative flow cytometry plots showing the frequencies of TIM-3^−^PD-1^+^ precursor exhausted T cells (Tpex) and TIM-3^+^PD-1^+^ terminally exhausted T cells (Tex) among donor OT-1 TILs. (G) Quantification and representative flow cytometry plots of TOX^+^TCF1^+^ progenitor-like cells and TOX^+^TCF1^−^ terminally exhausted cells among donor OT-1 TILs. (H) Quantification and representative flow cytometry plots showing the percentage of central memory–like (CD62L^+^CD44^+^) cells among donor OT-1 TILs. (I) Quantification and representative flow cytometry plots showing cytokine production, including IL-2, TNF-α, IFN-γ, and GZMB, in donor OT-1 TILs following ex vivo restimulation. Data in (A and B) are shown as mean ± SEM (n = 6), data in (D, E, F, G, H, and I) are shown as mean ± SEM (n = 3). Tumor growth curves (A) were analyzed by 2-way ANOVA. Survival curves (B) were compared using the log-rank (Mantel–Cox) test. All other comparisons were performed using an unpaired 2-tailed Student *t* test. *p < 0.050, **p < 0.010, ***p < .001. ANOVA = analysis of variance, BATF = basic leucine zipper ATF-like transcription factor, dLN = draining lymph node, GZMB = granzyme B, LNP = lipid nanoparticle, OVA = ovalbumin, TCR = T cell receptor, TIL = tumor-infiltrating lymphocytes, SEM = standard error of the mean, TIM-3 = T-cell immunoglobulin and mucin domain 3.

To directly evaluate the potency of our enhanced ACT approach, we utilized the B16-OVA melanoma model without prior chemotherapy-induced lymphodepletion or irradiation. The OT-1 TCR-T cell model against B16-OVA is recognized for its rapid progression, poor immunogenicity, and resistance to adoptive T-cell therapies, often resulting in incomplete or transient responses.^23,24^ To evaluate the impact of transient BATF OE on tumor growth and survival in vivo, C57BL/6 mice bearing established B16-OVA tumors received adoptive transfer of OT-1 CD8^+^ T cells transfected with either BATF mRNA–loaded SM-LNPs or empty control LNPs. BATF OE T cells suppressed tumor growth and significantly prolonged survival compared with controls (**Fig. 4A and B**). These results indicate improved anti-tumor efficacy with BATF OE.

To investigate the mechanisms underlying this superior tumor control, particularly with respect to T-cell accumulation and activity, we analyzed TILs and draining lymph nodes (dLNs) on day 5 post-transfer (day 12 after tumor inoculation), as shown in Figure 4C. BATF OE led to substantially greater accumulation of donor OT-1 cells within the tumor microenvironment. The frequency of donor (CD45.1^+^) OT-1 cells among total CD8^+^ TILs increased from 34.63 ± 3.76% (control) to 63.67 ± 6.82% (BATF) (**Fig. 4D**), corresponding to a 3.90-fold increase in absolute OT-1 numbers per gram of tumor (**Fig. 4D**). Consistent with enhanced T-cell activity, tumor size in the BATF OE group was significantly reduced at this time point (Figure S3A, https://links.lww.com/BS/A153), and a similar enrichment of donor OT-1 cells was observed in dLNs (Figure S3B, https://links.lww.com/BS/A153).

BATF OE OT-1 TILs also displayed enhanced proliferative activity, as evidenced by a 36.1% increase in the percentage of Ki67^+^ cells (**Fig. 4E**). Importantly, by day 5 post-transfer, BATF protein levels in both tumor-infiltrating and dLN-resident OT-1 cells had returned to baseline (Figure S3C and D, https://links.lww.com/BS/A153), confirming the transient nature of mRNA-LNP–mediated expression in vivo, consistent with the in vitro kinetics (**Fig. 2B**). These findings suggest that transient BATF OE promotes early T cell accumulation and proliferation in the tumor microenvironment.

To investigate how transient BATF OE influences T-cell differentiation, we examined phenotypic changes by analyzing memory- and exhaustion-associated markers. We found that transient BATF OE significantly reduced features of terminal exhaustion and instead increased the frequency of Tpex CD8^+^ T cell populations, including TIM-3^−^PD-1^+^ (25.17 ± 2.94% vs 46.37 ± 2.07%; **Fig. 4F** and Figure S3E, https://links.lww.com/BS/A153) and TOX^+^TCF1^+^ subsets (29.27 ± 3.03% vs 50.80 ± 1.07%; **Fig. 4G**), which are associated with preserved proliferative capacity and sustained anti-tumor immunity under chronic stimulation.^25^ Consistent with this shift toward a stem-like progenitor state, BATF OE also increased the proportion of central memory–like CD44^+^CD62L^+^ OT-1 TILs by 1.76-fold compared with controls (**Fig. 4H**). These observations are consistent with previous work showing that BATF restrains terminal exhaustion and promotes long-lived, memory-like CD8^+^ T cells in tumors.^10^

To investigate changes in cytokine production resulting from BATF OE within the tumor, we restimulated tumor-infiltrating T cells ex vivo and measured intracellular accumulation by flow cytometry. On restimulation with phorbol 12-myristate 13-acetate (PMA) and ionomycin in the presence of Brefeldin A, BATF OE OT-1 TILs exhibited enhanced polyfunctional cytokine responses (**Fig. 4I**). Frequencies of IL-2^+^ and tumor necrosis factor-α (TNF-α)^+^ cells were more than doubled among donor CD8^+^ TILs in the BATF group compared to controls, whereas interferon-γ (IFN-γ)^+^ and granzyme B (GZMB)^+^ populations remained comparable between groups, indicating better effector functions and expansion.

Collectively, these findings indicate that transient BATF mRNA delivery enhances the anti-tumor efficacy of TCR-T cells by boosting their accumulation, proliferative capacity, and polyfunctionality while restraining terminal exhaustion, which translates to superior survival outcomes.

### 3.5. BATF mRNA–LNP–transfected CAR-T cells improve control of hematologic malignancies in vivo

**Figure 5.**
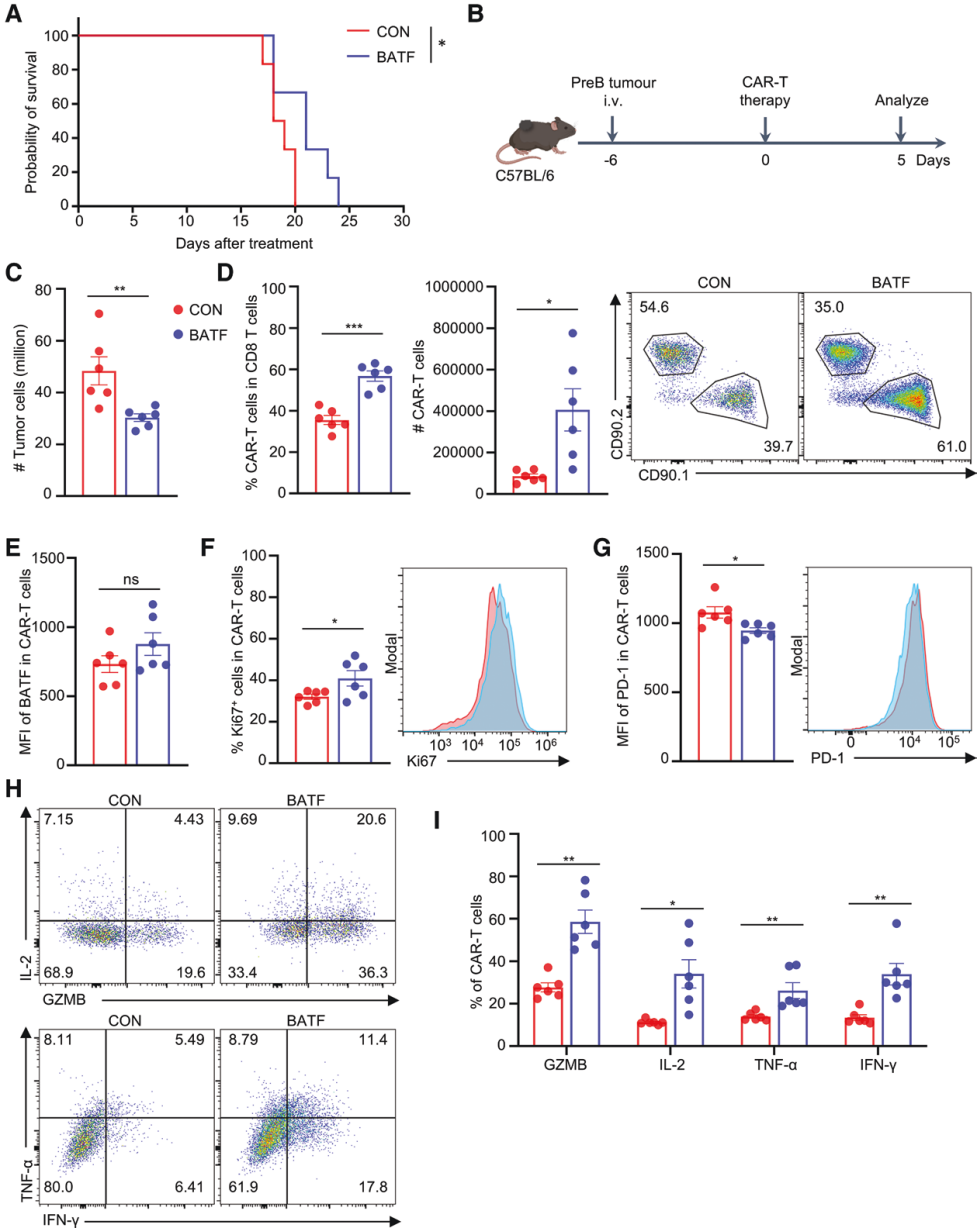
BATF mRNA–LNP–transfected CAR-T cells enhance clearance of hematologic malignancies in vivo. (A) Kaplan-Meier survival curves in disseminated hCD19^+^ pre-B ALL model treated with CAR-T cells transfected with control-LNP or BATF-LNP. (B) Schematic of the in vivo experiment analyzing leukemic burden and CAR-T cell responses in bone marrow and spleen at day 5 post-infusion. (C) Quantification of leukemic burden (absolute number of PE-Texas Red^+^ tumor cells) in the bone marrow at day 5. (D) Quantification and representative flow cytometry plots of CAR-T cell accumulation in bone marrow. Bar graphs show the percentage of CD90.1^+^ CAR-T cells among CD8^+^ T cells (left) and the absolute number of CD90.1^+^ CAR-T cells per femur (right). (E) Quantification of intracellular BATF MFI in CAR-T cells recovered from bone marrow at day 5. (F and G) Quantification of the percentage of Ki67^+^ CAR-T cells (F) and PD-1 MFI (G) on bone marrow-resident CAR-T cells at day 5. (H) Representative flow cytometry plots showing cytokine production (IL-2, TNF-α, IFN-γ, GZMB) in CAR-T cells. (I) Quantification of the percentages of bone marrow–resident CAR-T cells producing GZMB, IL-2, TNF-α, or IFN-γ following ex vivo restimulation. Data in (C, D, E, F, G, and I) are presented as the mean ± SEM (n = 6). Survival curves (A) were compared using the log-rank test. All other comparisons were performed using an unpaired 2-tailed Student *t* test. *p < 0.050, **p < 0.010, ***p < .001. ALL = acute lymphoblastic leukemia, BATF = basic leucine zipper ATF-like transcription factor, CAR-T = chimeric antigen receptor T cells, GZMB = granzyme B, IFN-γ = interferon-γ, LNP = lipid nanoparticle, TNF-α = tumor necrosis factor-α.

To further validate the enhancing effect on ACT in a hematological malignancy setting, we next investigated a highly aggressive leukemia model in the absence of any chemotherapy or irradiation preconditioning. The FMC63-based CD19 CAR-T cell therapy against hCD19^+^ Arf^−^^/^^−^ BCR-ABL pre-B ALL represents a rapidly fatal and therapy-resistant model, characterized by aggressive lymphoid leukemia development that limits complete responses.^20^ To evaluate the impact of transient BATF OE on CAR-T cell–mediated leukemia clearance in vivo, C57BL/6 mice were intravenously inoculated with disseminated hCD19^+^ pre-B ALL and subsequently treated with a single infusion of anti-human CD19 CAR-T cells. Donor CAR-T cells were transfected 48 hours earlier with BATF mRNA–loaded SM-LNPs or empty control LNPs. BATF OE CAR-T cells significantly delayed leukemia progression and prolonged survival compared with control CAR-T cells (**Fig. 5A**). Consistent with this improved therapeutic efficacy, BATF OE treatment substantially reduced leukemic burden on day 5 post-transfer (**Fig. 5B**). Absolute numbers of PE–Texas Red^+^ leukemic cells decreased by 37% in the bone marrow (**Fig. 5C**) and by 83% in the spleen (Figure S4A, https://links.lww.com/BS/A153), indicating superior early disease control.

To investigate whether this enhanced leukemia control was associated with augmented CAR-T cell expansion, we analyzed CAR-T cell accumulation in the bone marrow and spleen on day 5 post-infusion. BATF OE CAR-T cells exhibited significantly increased expansion in both compartments. The frequency of CAR^+^ cells among CD8^+^ T cells increased from 35.52 ± 2.18% to 56.77 ± 2.48% in the bone marrow (**Fig. 5D**) and from 22.50 ± 2.78% to 36.98 ± 4.19% in the spleen (Figure S4B, https://links.lww.com/BS/A153). Correspondingly, absolute CAR^+^ T cell numbers increased by 4.74-fold in the bone marrow and 2.47-fold in the spleen, indicating enhanced expansion upon tumor priming (**Fig. 5D** and Figure S4B, https://links.lww.com/BS/A153). Notably, although BATF protein expression returned to baseline by day 5 (**Fig. 5E** and Figure S4C, https://links.lww.com/BS/A153), the proportion of proliferating Ki67^+^ CAR^+^ CD8^+^ T cells remained higher in both the bone marrow (**Fig. 5F**) and spleen (Figure S4D, https://links.lww.com/BS/A153), indicating that transient BATF OE induced a sustained proliferative response in vivo.

Phenotypic analysis further revealed a marked modulation of inhibitory receptor PD-1 in BATF OE CAR-T cells. In both bone marrow and spleen, BATF OE CAR-T cells exhibited a consistent downregulation of the key exhaustion marker PD-1 MFI by approximately 12% in bone marrow and 27% in spleen (**Fig. 5G** and Figure S4E, https://links.lww.com/BS/A153). This reduction in PD-1 expression is critically associated with a diminished exhausted state and enhanced T cell function,^26^ supporting the observed superior therapeutic efficacy.

Next, we quantified the capacity of cytokine production of BATF OE CAR-T cells. CAR-T cells from the bone marrow and spleen were isolated and restimulated ex vivo to quantify GZMB, IL-2, TNF-α, and IFN-γ secretion by flow cytometry. In bone marrow, the frequencies of CAR^+^ CD8^+^ T cells producing GZMB, IL-2, TNF-α, and IFN-γ were all significantly increased, with GZMB production rising from 27.70% to 58.62%, IL-2 from 11.24% to 34.08%, TNF-α from 13.92% to 26.17%, and IFN-γ from 13.43% to 33.90% (**Fig. 5H and I**), indicating BATF OE CAR-T cells exhibited substantially augmented effector function. In the spleen, IL-2 and IFN-γ production were similarly enhanced, reaching 37.28% and 60.20%, respectively (Figure S4F and G, https://links.lww.com/BS/A153). Collectively, these data indicate that BATF OE enhanced the effector cytokine responses of CAR-T cells across multiple tissues.

Together, these results demonstrate that transient BATF OE enhances the therapeutic efficacy of CAR-T cells against leukemia. This improvement is mediated by promoted expansion, augmented polyfunctional cytokine production, and reduced exhaustion of CAR-T cells. Thus, LNP-mediated transient BATF modulation provides a practical strategy to augment CAR-T cell potency in hematologic malignancies.

## 4. DISCUSSION

In this study, we developed a transient, non-integrative BATF OE strategy by SM-102–based LNPs to improve the function of adoptively transferred CD8^+^ T cells. Using this platform, transient BATF OE via mRNA transfection resulted in elevated protein expression for 48 to 72 hours. This time-restricted BATF OE enhanced in vitro proliferation and cytotoxic activity while improving in vivo anti-tumor activity in both OT-1 TCR-T cells against B16-OVA melanoma and FMC63-based CD19 CAR-T cells against disseminated pre-B ALL. These results highlight the potential of transient transcription factor modulation to optimize ACT outcomes without permanent genetic alterations.

Our findings demonstrate that transient BATF OE significantly improves tumor control across distinct adoptive T cell therapy models, including TCR-T and CAR-T settings. In both models, transient OE of BATF promoted CD8^+^ T cell expansion and enhanced effector function, as evidenced by increased cytotoxicity and augmented effector cytokine production. These findings are in line with prior studies identifying BATF as a key regulator of CD8^+^ T cell fate. During acute responses, BATF acts as an essential checkpoint for initiating effector differentiation in naive CD8^+^ T cells, coordinating epigenetic remodeling and transcriptional programs in collaboration with partners like IRF4, Runx3, and T-bet.^8,27^ It promotes early proliferation, cytokine receptor expression (eg, IL-2R), and progenitor-to-effector transitions while delaying full effector commitment through incoherent feed-forward loops that balance activation and repression.^27,28^ BATF deficiency impairs effector expansion, memory formation, and responses to chronic viral infections or allografts, leading to reduced cytotoxicity, increased apoptosis, and retention of naive-like phenotypes.^9,22,28^ Moreover, studies overexpressing BATF in CAR-T cells have shown enhanced persistence, reduced exhaustion markers (eg, lower PD-1, TIM-3, LAG-3, TOX), increased polyfunctional cytokine production (eg, IL-2, TNF-α), and improved tumor control, often via IRF4-dependent mechanisms that preserve memory-like subsets and resist tumor-mediated inhibition.^10,11^ However, previous work has also shown that BATF can reinforce exhaustion under sustained stimulation, as its depletion in CAR-T cells has been linked to resistance against exhaustion, decreased PD-1 expression, and a shift toward central memory phenotypes, enhancing long-term anti-tumor activity.^12^ Notably, in our study, we found that transient BATF OE reduced exhaustion, as evidenced by reduced PD-1 expression, together with a shift toward progenitor-like (TOX^+^TCF1^+^) and memory-biased (CD44^+^CD62L^+^) states. By limiting BATF activity to a transient window, these effects were achieved without inducing terminal exhaustion, thereby avoiding the exhaustion associated with long-term BATF OE.

Our study also illustrates the utility of mRNA–LNP platforms for the transient modulation of transcription factor activity. Traditional viral methods enable durable expression but lack transient control and pose integration-related safety risks.^13^ In contrast, the SM-102–based LNP system we employed allows for a controlled and transient expression of BATF, avoiding these concerns while still achieving significant functional benefits. Moreover, this approach is compatible with scalable manufacturing,^29^ making it a promising strategy for enhancing T cell therapy in clinical settings. It is also important to consider the regulatory context of applying LNP-based mRNA delivery in ex vivo CAR-T cell manufacturing. Unlike mRNA vaccines, which focus on immunogenicity and broad population safety, CAR-T products are regulated as gene therapies, requiring strict oversight of manufacturing consistency, cellular potency, product quality, and long-term safety.^30^ Although LNPs enable transient, non-integrative expression, their use in CAR-T must ensure reproducible transfection, stable T-cell function, and minimal phenotypic impact for clinical translation.

Overall, our research demonstrates the value of transient BATF modulation via mRNA-LNPs for enhancing ACT. This approach improved therapeutic outcomes by promoting T-cell expansion, boosting cytokine production, and reducing exhaustion—key mechanisms that collectively enhanced efficacy in models of both solid and hematologic malignancies. By showing that transient BATF OE yields lasting benefits, our study provides insights into its context-dependent roles. Moreover, this method offers a safer, non-integrative alternative to permanent genetic modification, potentially improving the safety and efficacy profile of ACT. These advances hold promise for reducing relapse rates and improving outcomes for cancer patients.

## ACKNOWLEDGMENTS

This work was supported by the National Natural Science Foundation of China (52225310, 82341214, 32230055), the Distinguished Young Scholars of Tianjin (22JCJQJC00070), the CAMS Innovation Fund for Medical Sciences (CIFMS) (2023-I2M-2-007), the Non-profit Central Research Institute Fund of Chinese Academy of Medical Sciences (Grant No. 2022-RC320-01, 2021-RC310-011), the Shenzhen Science and Technology Program (JCYJ20240813114235046).

## AUTHOR CONTRIBUTIONS

All authors have made significant contributions to this work. J.W., D.D., and W.Z. conceived and designed the study and provided overall supervision and guidance. H.G. and K.W. performed the majority of experiments and data analysis. L.C., T.Y., F.Z., and H.L. assisted with experimental procedures and data interpretation. H.G., K.W., and T.Y. drafted the manuscript. All authors have read and approved the final manuscript and agree with its content.

## Supplementary Material
