# Supplementary material for: Enforced BATF expression via clinically approved LNPs enhances adoptive T-cell therapies
Source: Blood Sci. 2026 Jun 2;8(2):e00291. doi: 10.1097/BS9.0000000000000291 (PMC13232917; doi:10.1097/BS9.0000000000000291)
Supplement: Supplementary file 1 [file bs9-8-e00291-s001.pdf]

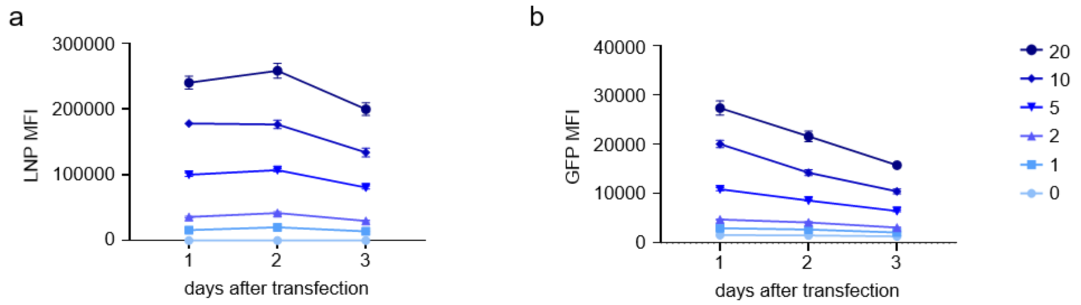

Figure S1 | Dose-dependent uptake and transgene expression of SM-LNPs in mouse CD8⁺ T cells.
A-B, GFP MFI **(A)** and Dose-dependent DIL MFI (LNP uptake) **(B)** expression in mouse CD8 T cells at days 1, 2, and 3 post-transfection with SM-LNPs at the indicated mRNA concentrations (0–20 µg/mL). Data are presented as mean ± s.e.m. (n=3).

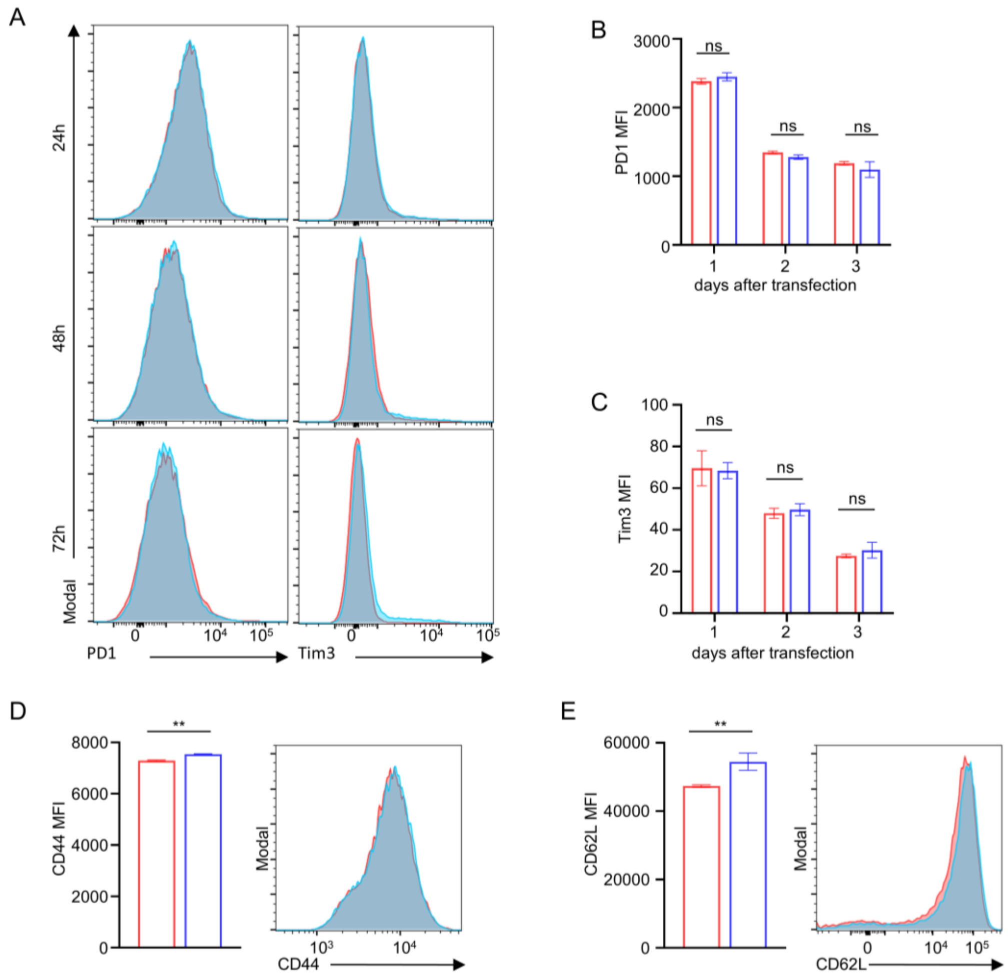

Figure S2 | Exhaustion and memory markers in BATF-OE T cells.

A-C, Representative flow cytometry histograms (**A**) and quantification of PD-1 (**B**) and TIM-3 (**C**) MFI in T cells 24 h post-transfection.

D-E, MFI of CD44 (**D**) and CD62L (**E**) in T cells 24 h post-transfection.

Data are presented as mean ± s.e.m. (n=3). P values were determined by unpaired two-tailed Student's t-test. **P < 0.01.

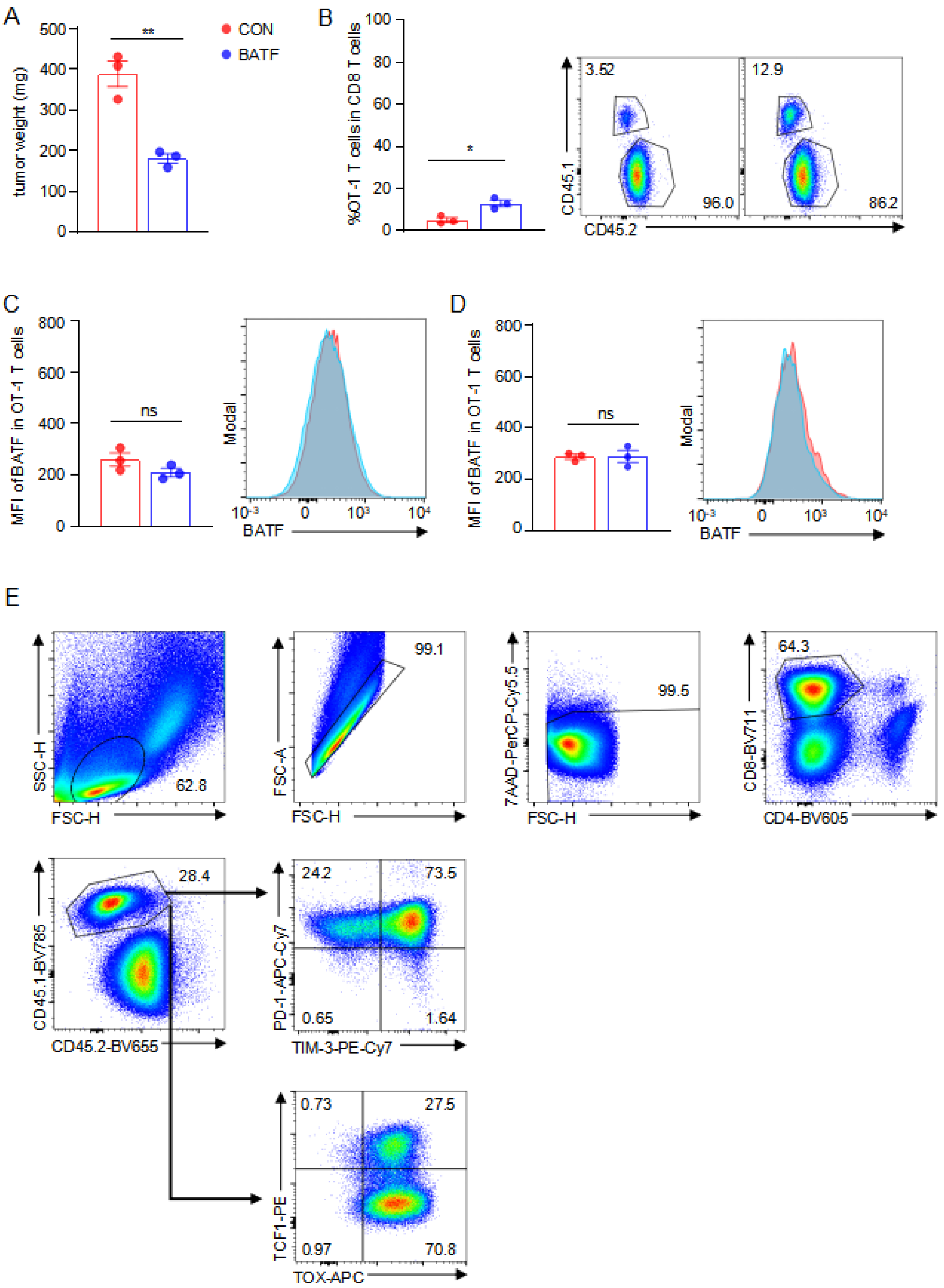

Figure S3 | In vivo tumor weights and BATF kinetics in solid tumor model.

A, Tumor weights at day 5 post-transfer.

B, Quantification of donor OT-1 T cell infiltration in dLNs Bar graphs show the percentage of donor cells among total CD8$^+$.

C, Quantification of BATF MFI in donor OT-1 TILs at day 5 post-transfer.

D, Quantification of BATF MFI in donor OT-1 in dLNs at day 5 post-transfer.

Data are presented as mean ± s.e.m. (n=3). *P* values were determined by unpaired two-tailed Student's t-test. *$P < 0.05$, **$P < 0.01$.

E, Representative gating strategy for analysis of Tpex CD8$^+$ T cell populations. CD8$^+$ T cells were analyzed for TIM-3$^-$PD-1$^+$ and TOX$^+$TCF1$^+$ subsets by flow cytometry.

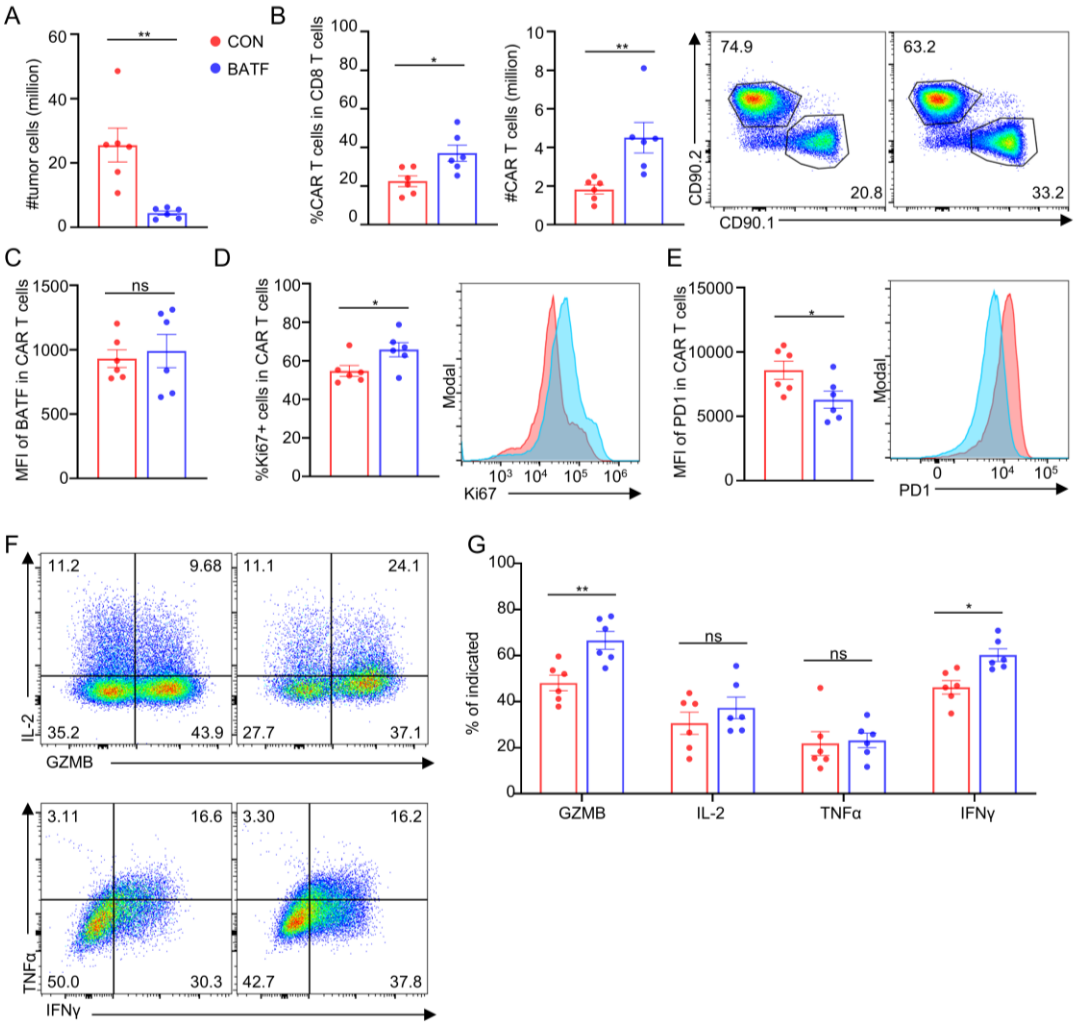

Figure S4 | Spleen immune profiling and CAR-T cell functional analysis in the B-ALL leukemia model.

A, Absolute number of leukemic cells in spleen at day 5.

B, Quantification and representative flow cytometry plots of CAR-T cell accumulation in spleen. Bar graphs show the percentage of CD90.1$^+$ CAR-T cells among CD8$^+$ T cells (left) and absolute numbers of CD90.1$^+$ CAR-T cells in spleen (right).

C, Quantification of intracellular BATF MFI recovered from spleen at day 5.

D, E, Quantification the percentage of Ki67$^+$ (**D**) and PD-1 MFI (**E**) on spleen-resident CAR-T cells.

F, Representative flow cytometry plots showing cytokine production (IL-2, TNF-α, IFN-γ, GZMB) in CAR-T cells.

G, Quantification of the percentage of cytokine-producing CAR-T cells in spleen after restimulation.

Data are presented as mean ± s.e.m. (n= 6). *P* values were determined by unpaired two-tailed Student's t-test. *$P < 0.05$, **$P < 0.01$.
